# Uptake of Genital Mucosal Sampling in HVTN 097, a Phase 1b HIV Vaccine Trial in South Africa

**DOI:** 10.1371/journal.pone.0112303

**Published:** 2014-11-17

**Authors:** Erica Maxine Lazarus, Kennedy Otwombe, Tania Adonis, Elaine Sebastian, Glenda Gray, Nicole Grunenberg, Surita Roux, Gavin Churchyard, Craig Innes, Fatima Laher

**Affiliations:** 1 Perinatal HIV Research Unit, Faculty of Health Sciences, University of the Witwatersrand, Johannesburg, South Africa; 2 The Aurum Institute, Klerksdorp, South Africa; 3 Desmond Tutu HIV Foundation, University of Cape Town, Cape Town, South Africa; 4 Fred Hutchinson Cancer Research Center, Seattle, Washington, United States of America; University of Missouri-Kansas City, United States of America

## Abstract

Because sexual transmission of HIV occurs across mucosal membranes, understanding the immune responses of the genital mucosa to vaccines may contribute knowledge to finding an effective candidate HIV vaccine. We describe the uptake of rectal secretion, cervical secretion and seminal mucosal secretion sampling amongst volunteers in a Phase 1b HIV vaccine trial. Age at screening, gender, study site and the designation of the person conducting the informed consent procedure were collected for volunteers who screened for the HVTN 097 study. A total of 211 volunteers (54% female) were screened at three sites in South Africa: Soweto (n = 70, 33%), Cape Town (n = 68, 32%) and Klerksdorp (n = 73, 35%). Overall uptake of optional mucosal sampling amongst trial volunteers was 71% (n = 149). Compared to Cape Town, volunteers from Soweto and Klerksdorp were less likely to consent to sampling (Soweto OR 0.08 CI: 0.03–0.25 p<0.001 and Klerksdorp OR 0.13 CI: 0.04–0.41 p = 0.001). In contrast, volunteers over 25 years of age were 2.39 times more likely to consent than younger volunteers (CI: 1.13–5.08, p = 0.02). Further studies are required to better understand the cultural, demographic and sociobehavioral factors which influence willingness to participate in mucosal sampling in HIV prevention studies.

**Trial Registration:**

ClinicalTrials.gov: NCT02109354

## Introduction

Despite the upscale of HIV prevention strategies like male medical circumcision, there were an estimated 2.1 million new HIV infections globally in 2013 [1]. In South Africa, the country with the highest number of people living with HIV, where heterosexual HIV transmission is predominant, there were an estimated 340,000 new infections in 2013 alone [1].Sexual transmission remains the driver of the AIDS epidemic, especially in Sub-Saharan Africa where the burden is highest [1]. Developing a multifaceted package of prevention tools to curb the epidemic is crucial, and an efficacious HIV vaccine will have a major contribution toward reaching the UNAIDS goal of zero new infections [2].

The landmark RV 144 Thai trial which investigated the use of a recombinant canarypox vector vaccine, ALVAC, in combination with recombinant glycoprotein 120 subunit vaccine, AIDSVAX, was the first HIV vaccine trial to show efficacy [3]. The HIV Vaccines Trials Network (HVTN) 097 trial is a Phase 1b study designed to assess the safety and immunogenicity of the HIV Clade B/E Thai regimen vaccines in a South African population where the predominant HIV strain is Clade C.

Because sexual transmission of HIV occurs across mucosal membranes, much work has been done to elucidate the innate factors central to genital mucosal immunity [4],[5]. Several mucosal innate immune factors with in vitro anti-HIV properties, such as the immune proteins Secretory Leukocyte Protease Inhibitor (SLPI), Trappin-2, Lactoferrin and Defensins α and β, have been explored as possible immune correlates of protection in vivo in at-risk populations including men who have sex with men, highly exposed persistently seronegative women and HIV exposed infants [4]. As research continues, understanding the mucosal immune response to vaccines in development may contribute knowledge to finding an effective candidate [6],[7]. Preclinical studies in rhesus macaques have shown that systemic vaccination against Simian Immunodeficiency Syndrome (SIV) induced SIV specific immune responses in mucosal tissues, which were associated with decreased cellular viral loads [8],[9] One of the primary aims of RV 306, a follow-up study to the RV144 trial currently recruiting participants, is to characterise vaccine induced immune responses to the RV144 regimen in systemic and mucosal compartments [10]. Similarly, HVTN 097 seeks to evaluate the HIV specific immune correlates of protection identified in RV144 in the mucosal compartment. Therefore various combinations of optional mucosal sampling of rectal, cervical and seminal compartments may be performed on participants in HIV vaccine trials which investigate mucosal responses.

Rectal secretion, cervical secretion and semen sampling are the current methods of mucosal sampling validated by the HVTN mucosal immunology group [11],[12]. These are benign procedures with minimal side effects, mainly minor discomfort. Other side effects of cervical secretion sampling are similar to those associated with Pap smear testing including minor bleeding, pain and abdominal cramping but these are uncommon. Rectal secretion sampling may also result in minor bleeding although this is also seldom seen. While all procedures may cause embarrassment or anxiety, procedures are optional and consent can be withdrawn at any time without affecting participation in the study.

Although the literature supports the expressed willingness of communities to participate in hypothetical preventive HIV vaccine trials [13]–[18], to the authors' knowledge, no literature to date describes the willingness of those who screen for such studies to participate in mucosal sampling procedures. We describe the actual written willingness of volunteers who were screened for HVTN 097 to undergo optional rectal secretion, cervical secretion and seminal mucosal secretion sampling.

## Methods

**Figure 1:**
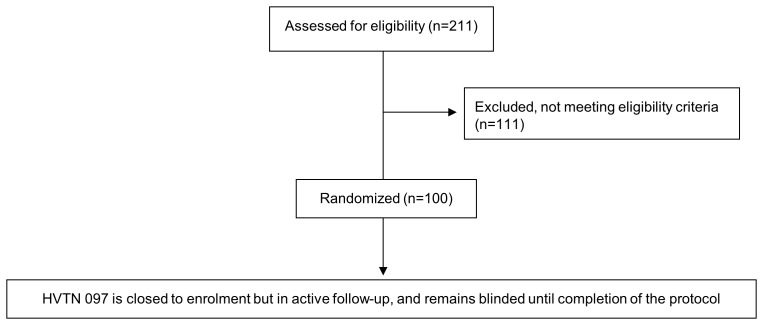
Flow diagram for HVTN 097 screening and enrollment.

This cross-sectional study was conducted using non-trial data collected from the three South African sites which screened participants for HVTN 097 located in Cape Town, Western Cape province (HIV prevalence 5.0%); Klerksdorp, North West province (HIV prevalence 13.3%) and Soweto, Gauteng province (HIV prevalence 12.4%) [19]. Screening for HVTN 097 took place between June 2013 and November 2013. As shown in Figure 1, a total of 211 volunteers were screened and 100 were enrolled per protocol. Enrolled participants remained in active follow-up throughout data collection and analysis for this report, which occurred between December 2013 and February 2014.

HVTN 097 was a phase 1b randomized double blind placebo controlled clinical trial to evaluate the safety and immunogenicity of the RV144 vaccine regimen in healthy, HIV-1 uninfected adult participants at low risk for acquiring HIV at three sites in South Africa: Soweto, Cape Town and Klerksdorp. Low risk was defined as being sexually abstinent, or in a mutually monogamous relationship with a partner of known HIV-uninfected status, or having one partner believed to be HIV-uninfected with whom he/she regularly used condoms for vaginal or anal intercourse, during the 12 months prior to enrolment into HVTN 097. Volunteers for the study provided written informed consent simultaneously for the study and optional mucosal sampling procedures prior to beginning screening processes. At the time of consent, all volunteers were given the option to undergo rectal secretion and/or cervical secretion (female)/seminal (male) mucosal secretion sampling at three time points during the study: at enrolment to provide baseline values of mucosal immunity, midway (Day 210) and toward study completion (Day 394) to provide information on the durability of vaccine-induced mucosal immunity. Volunteers could refuse consent to any or all of the procedures without affecting their eligibility to enrol in the study.

The Participant Information Leaflet described risks (e.g. short-lived discomfort and minor bleeding), benefits (testing and treatment as required for sexually transmitted infections (STIs)) and the procedures involved in sampling. Rectal secretion sampling involved anoscope insertion into the rectum and absorption of fluids by sponge for 5 minutes. For cervical secretion sampling, vaginal speculum insertion was followed by sponge placement into the endocervix. Rectal and cervical secretion sampling were performed by a study physician. Semen sampling involved masturbation into a container, which could be done at the clinical research site or at home depending on volunteer preference.

Eligibility for mucosal sample collection included absence of local lesions and no sexual intercourse or use of topical products within 48 hours of sampling. For cervical secretion sampling, participants could not be menstruating, had to have a negative pregnancy test and must have had a normal Pap smear test result within the 3 years prior to the first sampling procedure. Female volunteers who had not had a recent Pap smear but agreed to cervical secretion sampling had Pap smears performed on site (Soweto and Cape Town) or were referred to nearby facilities (Klerksdorp).

Information was collected by site coordinators who reviewed volunteer informed consent documents obtained in local languages. Birth date and sex at birth demographics were collated from volunteer records. Qualitative sociobehavioral data was not collected as this was not included in the study protocol. We collated the following information amongst all volunteers who consented to screen for the HVTN 097 trial regardless of their final study or sampling eligibility status, or their decision on whether or not to participate in any mucosal sampling procedures: volunteer age, sex at birth, research site and designation of staff conducting consent.

### Statistical Analysis

Age was analysed descriptively as a continuous variable and then categorised into the age-groups 18–24, 25–30 and >30 years. Frequencies and their proportions were determined for all categorical variables, and presented overall and by sex at birth. Comparison of categorical variables by sex at birth was done using the chi-square test of proportions.

Predictors of consenting to any mucosal sampling strategy were determined using univariate and multivariate logistic regression. Statistical analysis assumed a two-sided 5% significance level and was performed using SAS Enterprise Guide version 5.1 (Analysis Software Institute, Cary, NC, USA).

### Ethical Considerations

HVTN 097 received approval from the University of the Witwatersrand Human Research Ethics Committee and the University of Cape Town Ethics Committee.

## Results

A total of 211 volunteers (54% female) were screened at the three sites: Soweto (n = 70, 33%), Cape Town (n = 68, 32%) and Klerksdorp (n = 73, 35%). The median age was 22.6 years (IQR: 20.4–26.9), and the majority were consented by trained lay counsellors (n = 196, 93%). Soweto enrolled significantly more males than females, whilst the opposite was true of Cape Town (Table 1).

**Table 1:** Demographics of all volunteers screened for the HVTN 097 trial.

Variable	Total sample (n = 211)	Females (n = 114)	Males (n = 97)	p-value
**Median Age (IQR) in years**	22.6 (20.4–26.9)	22.8 (20.5–27.1)	22.6 (20.4–26.6)	0.87
**Age**				
18–24 years (%)	132 (63)	71 (62)	61 (63)	0.93
25–30 years (%)	57 (27)	31 (27)	26 (27)	0.95
>30 years (%)	22 (10)	12 (11)	10 (10)	0.96
**Site**				
Soweto (%)	70 (33)	26 (23)	44 (45)	0.001
Klerksdorp (%)	73 (35)	40 (35)	33 (34)	0.87
Cape Town (%)	68 (32)	48 (42)	20 (21)	0.001
**Designation of person conducting informed consent**				
Counsellor (%)	196 (93)	112 (98)	84 (87)	0.001
Clinician (Doctor/Nurse) (%)	15 (7)	2 (2)	13 (13)	-
**Enrolled into study**				
Yes (%)	100 (47)	65 (57)	46 (47)	0.16
No (%)	111 (53)	49 (43)	51 (53)	-
**Consented to**				
Any sampling (%)	149 (71)	90 (79)	59 (61)	0.004
* Rectal secretions only (%)*	6 (3)	1 (0.8)	5 (5)	-
* Cervical secretion or Semen only (%)*	63 (30)	36 (32)	27 (28)	0.55
* Rectal secretion +Cervical secretion/Semen (%)*	80 (38)	53 (46)	27 (28)	0.005
None (%)	62 (29)	24 (21)	38 (39)	0.004

Twenty nine percent (62/211) of volunteers who screened for participation in HVTN 097 declined all mucosal sampling procedures. Of the 100 volunteers eventually enrolled into HVTN 097, 68% (n = 68) had provided consent for mucosal sampling. This was not significantly different to the 73% (n = 81) of screening failures who had provided consent for mucosal sampling (p = 0.43). Uptake of combination sampling was not significantly different to any single sampling method alone (p = 0.37). The proportion of females consenting to procedures that included cervical secretion sampling was similar between the two sites (Soweto and Cape Town) that performed in-house Pap smear testing versus the site (Klerksdorp) which referred out for Pap (57/74 (77%) vs. 32/40 (80%); p = 0.71). Although more women than men consented to mucosal sampling procedures (n = 90, 79% vs. n = 59, 61%; p = 0.004), sex at birth was not identified as a predictor for sampling consent in multivariate analysis. However, 47% (n = 54) of females consented to rectal secretion sampling and 77% (n = 88) to cervical secretion sampling. And for males, 33% (n = 32) consented to rectal secretion sampling and 55% (n = 53) to semen sampling.

In the multivariate logistic regression controlling for the designation of person conducting informed consent shown in table 2, those between 18–24 years old (OR: 0.418, CI: 0.197–0.888), and those recruited in Soweto (OR: 0.078, CI: 0.025–0.250), and Klerksdorp (OR: 0.133, CI: 0.042–0.414) had a lower odds of consenting to any mucosal sampling strategy.

**Table 2:** Predictors of consenting to mucosal secretion sampling amongst volunteers screened for HVTN 097.

Variable	Univariate		Multivariate	
	OR* (95% CI**)	P-value	AOR† (95% CI**)	P-value
**Age**				
18–24	Ref		Ref	
≥25	2.65 (1.35–5.23)	0.005	2.39 (1.13–5.08)	0.02
**Gender**				
Male	0.41 (0.23–0.76)	0.004	0.52 (0.26–1.04)	0.06
Female	Ref		Ref	
**Site**				
Soweto	0.07 (0.02–0.20)	<0.001	0.08 (0.03–0.25)	<0.001
Klerksdorp	0.13 (0.04–0.39)	<0.001	0.13 (0.04–.41)	0.001
Cape Town	Ref		Ref	
**Designation**				
Counsellor	Ref		Ref	
Clinician	0.60 (0.20–1.76)	0.35	2.22 (0.66–7.40)	0.20

*OR  =  Odds Ratio.

**95% CI  =  95% confidence interval.

† AOR  =  Adjusted Odds Ratio.

## Discussion

To our knowledge, this is the first paper describing consent to optional mucosal sampling procedures in HIV vaccine trials. Overall uptake of optional mucosal sampling amongst HVTN 097 vaccine trial volunteers was 71% despite the personal and invasive nature of the procedure. Our study finds that there were regional differences in the uptake of mucosal sampling at the various sites, suggesting different sociocultural preferences, differences in local site staff interaction with volunteers, or differences in consenting procedures, for example group discussions prior to individual consent and degree of privacy in the rooms used to discuss information pamphlets. Older volunteers were significantly more likely to consent to mucosal procedures than younger individuals.

Though work has not been done to explore the motivators for consenting to mucosal sampling, previous studies investigating expressed willingness to participate (WTP) in hypothetical HIV vaccine trials may shed some light. In a previous study at the Soweto site, it was demonstrated that less exposure to social stressors was associated with higher expressed WTP in HIV biomedical prevention trials in adolescents aged 16–18 years [15]. At the same site, adolescents rated the following benefits as “very important” in expressing a WTP in a hypothetical trial: receiving current information about HIV research, doing something to honor people who have HIV or have died of AIDS, obtaining free HIV counseling and testing, possibility of protection against HIV, and improving motivation to avoid risky behavior. Unlike our study which measured actual consent to a procedure, this adolescent vaccine trial preparedness study showed no significant differences in expressed WTP in a hypothetical trial by sex at birth [20]. In one study in Cape Town, although more adults reported expressed WTP than adolescents at the start of the study, this difference was eliminated after attending two educational workshops on HIV, vaccines and vaccine trials [16]. This corroborated other Cape Town data which demonstrated that increasing knowledge about HIV vaccines is associated with higher expressed WTP in hypothetical HIV vaccine trials [14].

Another Cape Town study showed that five factors affected expressed WTP in hypothetical vaccine trials: personal costs, safety and convenience, stigmatization, personal gains, and social approval and trust [17]. The regional differences of consenting to mucosal sampling shown in our study may be explained by some of these factors due to variations in community characteristics.

There may also be factors related to mucosal sampling which are different to simply participating in a vaccine trial which could affect mucosal sampling uptake, for example mistrust of staff handling these sensitive samples or psychosocial discomfort.

Although our study is not able to assess whether consenting to mucosal sampling procedures translates into actual participation in these procedures, a vaccine preparedness study conducted in the United States of America found that significantly more participants who expressed definite willingness to participate in a hypothetical vaccine trial were eventually enrolled compared to those who expressed probable willingness or probable/definite unwillingness. However, ultimately only 20% of those participants who had stated hypothetical willingness to participate did actually enroll in the HIV vaccine trial indicating that willingness to participate may overestimate actual participation [21].

STI testing is not routinely available in the public health sector in South Africa. STI treatment is only provided to symptomatic persons who present for care using a World Health Organization based syndromic management approach, which involves the use of multiple drugs to cover the most likely causes of infection [22],[23]. Although this study enrolled individuals whose sexual behavior was considered to be low risk for acquiring HIV and therefore also other STIs, access to free STI testing and specific treatment may have played a role in promoting uptake of sampling.

For women in our study, one of the benefits of consenting to cervical secretion procedures was free access to cervical cancer screening by Pap smear. In the South African National Guideline for Cervical Cancer Screening, free public sector Pap smear testing is available to HIV-uninfected women only from the age of 30 years and then every 10 years thereafter to a maximum of three lifetime tests, unless abnormalities warranting further investigations are detected [24].

Cultural factors may play a role in the uptake of semen sampling. Various studies noted male anxiety around masturbation [25]–[27], which would pose a barrier to semen sampling in vaccine trials. In Sri Lanka for example semen loss by masturbation or nocturnal emission was perceived as detrimental to mental and physical health [27]. However, the authors are not aware of any similar research in the South African context.

A strength of our study is that, unlike many other studies referenced, it does not describe expressed WTP but rather measures factors associated with actual consent to procedures in South African sites. However, owing to the cross-sectional nature of the study, we are unable to account for consent withdrawal over time for which longitudinal data would be required. A limitation of our study is that we did not collect reasons which motivate or impede expressed WTP.

Further studies are required to better understand the cultural, demographic and sociobehavioral factors which influence willingness to participate in mucosal sampling in HIV prevention studies.
